# Inside- and Outside-Coated PANI and/or PIN-TiO_2_ Nanotubes for Enhanced Photocatalytic Degradation of 4-Nitrophenol in Wastewater

**DOI:** 10.1021/acsomega.4c08137

**Published:** 2024-12-16

**Authors:** Seyed Mohammad Matin Ahmadi, Afsanehsadat Larimi, Ali Akbar Asgharinezhad, Farhad Khorasheh, Cyrus Ghotbi

**Affiliations:** † Department of Chemical and Petroleum Engineering, Sharif University of Technology, Tehran 1458889694, Iran; ‡ School of Engineering and Applied Sciences, Department of Chemical Engineering, Swansea University, Swansea SA1 8EN, Wales, U.K.; § Chemistry and Process Research Department, Niroo Research Institute (NRI), Tehran 14665517, Iran

## Abstract

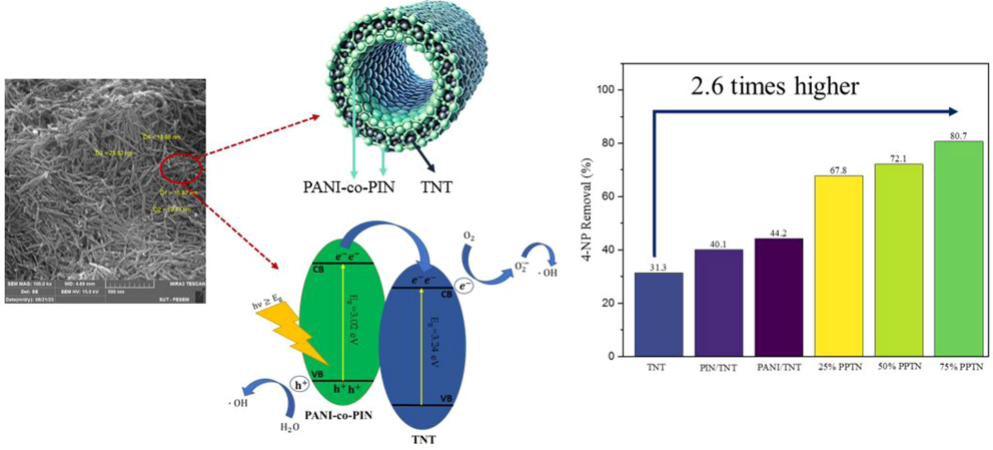

We present a novel approach for enhancing photocatalytic efficiency by developing polyaniline (PANI) and polyindole (PIN)-coated TiO_2_ nanotubes (TNT) through a combination of chemical oxidation and hydrothermal processes. The PANI–PIN coating was systematically applied to both the internal and external surfaces of the nanotubes to enhance the photocatalytic active sites and optimize pollutant adsorption. The dual-coated structure enhances the interaction with pollutants, facilitating a more efficient degradation of 4-nitrophenol (4-NP) when exposed to visible light. Thorough characterization through X-ray diffraction (XRD), Fourier-transform infrared (FTIR), field emission scanning electron microscopy (FE-SEM), transmission electron microscopy (TEM), energy-dispersive X-ray (EDX), N_2_-physisorption, transient photocurrent, diffuse reflectance spectroscopy (DRS), and photoluminescence (PL) validated the exceptional structural and optical properties of the composite. The PANI/PIN polymer coating effectively inhibited electron–hole recombination, leading to a notable enhancement in photocatalytic performance. Among the tested composites, the formulation consisting of 75% PANI and 25% PIN demonstrated remarkable performance, achieving a degradation rate of 99.46% for 4-NP in only 120 min of exposure to visible light. The impressive efficiency stems from its extensive surface area (255.3 m^2^/g), efficient charge separation, minimized band gap (2.77 eV), and improved light absorption. Moreover, the composite demonstrated remarkable recyclability, preserving its catalytic activity across five cycles without any decline in performance. These results demonstrate the strong potential of 75%PPTN as a promising photocatalyst for environmental remediation.

## 1 Introduction

One of the most paramount problems faced by human beings is to have access to potable water easily and at any time since it is reckoned as the most critical of all natural resources for maintaining life on earth. Organic compounds, heavy metals, pharmaceutical compounds, pesticides, etc., are some of the pollutants that exist in the environment. Among organic pollutants, phenolic compounds are highly soluble in water and produced by extensive industrial processes. Amid reported phenolic compounds, nitrophenols (NPs), which contain benzene rings, nitro and hydroxyl groups are extensively used for the production of paint, preservatives, pesticide, pharmaceutical, herbicides etc., and are found to be highly toxic and carcinogenic that makes them tremendous threat not only to human health also to aquatic species and environment.^1−3^

Among its derivatives, 4-Nitrophenol (4-NP) has been identified as a priority pollutant, and due to its high stability, purifying 4-NP-contaminated wastewater is problematic. There is a very low level of photochemical transformation of 4-NP in aqueous aerated solutions.^4,5^ Hence, it has been difficult to conquer an applicable method for thriftily removing toxic pollutants from wastewater like 4-NP.^6^ Contrary to conventional treatment systems, heterogeneous photocatalysis techniques have proved to mineralize organic pollutants thoroughly under mild circumstances like ambient temperature and pressure.^7^

The most common metal oxide semiconductors are TiO_2_, CeO_2_, ZnO, Fe_2_O_3_, V_2_O_5_, CuO, In_2_O_3_, and others.^8−11^ Among the most common metal oxide semiconductors, TiO_2_ and ZnO are best known.^12−14^ Most of the attributes associated with TiO_2_ have focused on aspects such as its resilience when exposed to light, ease of use, lack of harmful properties, efficient function at lower temperatures, minimal energy usage, outstanding performance in promoting photocatalysis, suitable resistance to chemical changes, inability to dissolve in water, and preventing the formation of undesirable secondary substances.^15,16^ This confluence of factors establishes it as an exceptionally impactful photocatalyst.^1,16^

However, this remarkable photocatalyst does have two inevitable limitations. The initial drawback is its band gap, approximately 3.2 eV (for the anatase phase), rendering it incapable of initiating photocatalytic reactions using solar light.^6,16^ The second one is the elevated potential for recombination of electron–hole pairs, formed by photons.^15−17^

To address the deficiencies of TiO_2_, the implementation of conductive polymers (CPs) has been proposed. Conducting polymers, including polyaniline (PANI), polyindole (PIN), polypyrrole (PPy), and polythiophene (PTh), together with their derivatives, are utilized in environmental remediation, energy storage, photosensitization, and biological applications,^18−20^ and so on. They not only diminish the challenging band gap but also enhance electron–hole transport capability.^21,22^ Notable features of PANI encompass improved charge carrier separation, increased quantities of hydroxyl free radicals, and enhanced photoactivity under standard light, especially with decreased energy absorption.^23,24^ Moreover, PANI preferentially adsorbs negatively charged species due to the positive charge present on its surface, facilitating electrostatic attraction to anions.^19,25^ Likewise, PIN may be readily manufactured via a simple chemical oxidation procedure and demonstrates thermal stability, modest degradation rates, elevated redox activity, and tunable conductive characteristics relative to other conductive polymers.^26^ The complementary properties of PANI and PIN within the PANI–PIN/TiO_2_ nanocomposite presents a compelling approach for improving photocatalytic performance, leveraging the synergistic characteristics of these two conductive polymers. PANI is recognized for its exceptional conductivity, straightforward synthesis process, and redox activity, positioning it as a prime candidate for use in supercapacitors and catalysis.^27,28^ Meanwhile, PIN provides enhanced thermal stability, chemical resilience, and significant redox activity, thereby complementing the characteristics of PANI.^28^ Research has shown that integrating PANI with PIN enhances charge transfer, electron mobility, and surface area, as reflected in the improved electrochemical performance of PANI–PIN composites in supercapacitors.^27,28^ Moreover, the uneven surface structure resulting from the copolymerization of PANI and PIN improves the adsorption of pollutants, which is essential for photocatalytic degradation.^27^ The PANI–PIN/TiO_2_ nanocomposite utilizes the stability of PIN and the conductivity of PANI to enhance photocatalytic activity, addressing a significant gap in the use of conductive polymers for environmental remediation. This innovative combination shows promise not only for energy storage but also for improving the efficiency of pollutant degradation when exposed to visible light.^27,28^

Karegar et al.^29^ presented a simple and cost-effective approach for synthesizing a magnetic nanocomposite based on polyindole, enhanced with silver nanoparticles (Fe_3_O_4_–PIN-Ag) at room temperature, without the need for any reducing agents or stabilizers. In this environmentally friendly synthesis process, polyindole, as a CP, serves a dual function: it not only reduces silver ions (Ag^+^) but also stabilizes the Ag NPs on the polymer shell surface. Sambaza et el.^30^ degraded Bisphenol A (BPA) by PANI-wrapped TiO_2_ nanorods, which prevailed 99.7% degradation after 80 min in optimized condition. In a separate investigation, Kumar et al.^31^ conducted research on nanocomposites involving TiO_2_, TiO_2_/PANI, and TiO_2_/PANI/GO to facilitate the photodegradation of Rose Bengal and Thymol blue dyes using visible light. Under specific conditions of 180 min of exposure, an initial dye concentration of 25 mg/L, a photocatalyst dosage of 1600 mg/L, and a neutral pH environment, they achieved significant degradation rates. Specifically, Thymol Blue and Rose Bengal dyes were effectively degraded by 85–99, 60–97, and 10–20%, respectively.

Furthermore, Cui et al.^32^ utilized thermoplastic polyurethane (TPU)/TiO_2_/PANI composite, which was prepared via the electrospinning method, in a photocatalytic reaction to reduce Cr(VI) in wastewater. According to their findings, they successfully purified water contaminated with Cr(VI), achieving a remarkable 99% reduction in this pollutant.

By the same token, Singh et al.^33^ utilized PANI-enwrapped CoFe_2_O_4_/g-C_3_N_4_ ternary nanocomposite to reduce organic pollutants. They depicted that after 40 min, this ternary nanocomposite could achieve a 96.2% reduction in 4-NP in optimal conditions. Yadav et al.^34^ applied the wet impregnation method to synthesize vanadium porphyrin complex-TiO_2_ to reduce 4-NP. This catalyst demonstrated a reduction of the pollutant by up to 99%, compared to the pure TiO_2_, which achieved 87% degradation.

A significant challenge in wastewater treatment lies in the restricted efficiency and stability of traditional photocatalysts. This study presents a unique methodology utilizing polyaniline-*co*-polyindole (PANI–PIN)/TiO_2_ nanotubes (referred to as PPTN) that are coated on both the inside and outside, synthesized through a straightforward and economical hydrothermal process. The reasoning for applying a coating to both the interior and exterior surfaces of the TNT is to optimize the photocatalytic active sites and improve pollutant adsorption. Applying a coating to the inner surface enhances the interaction with pollutants as they diffuse through the nanotubes, whereas the outer coating effectively traps pollutants on the exterior, resulting in improved degradation. The dual-coated structures present an improved surface area, enhanced light absorption, and effective charge separation, leading to a notable increase in photocatalytic efficiency. Although earlier investigations have examined a range of photocatalysts, we believe this is the inaugural instance of utilizing PPTN with distinctive inside–outside coatings and nanotube structures for the purpose of wastewater treatment. The main aim was to examine the degradation of 4-NP when exposed to visible light. This study further optimized essential variables that influence the efficiency of pollutant removal, such as pH, catalyst dosage, pollutant concentration, oxidant volume, and reaction time. Through an investigation of the synergistic effects of PANI and PIN in the distinctive PPTN formulation, we seek to deepen the comprehension of CPs regarding their synthesis, effectiveness, and reaction dynamics, thereby improving their applicability in water purification technologies.

## 2 Experimental Section

### 2.1 Materials

All chemicals including iron(III) chloride (FeCl_3_·6H_2_O), aniline, indole, sodium hydroxide (NaOH), titanium dioxide (TiO_2_), hydrochloric acid (HCl), 4-NP, ethanol, acetone, hydrogen peroxide (H_2_O_2_, 30%) and distilled water were purchased from Merck Co (Germany).

### 2.2 Instruments and Characterization

A variety of analytical methods were used to characterize the samples. To ascertain the composition of the crystal phase, X-ray diffraction (XRD, PANalytical XPert Pro MPD) at 40 kV and 40 mA was performed using Cu–Kα radiation (λ = 1.5406 Å). The analysis of functional groups of photocatalysts was performed using Fourier transform infrared spectroscopy (FTIR, PerkinElmer) throughout the 400–4000 cm^–1^ range. The examination of particle size and morphology was conducted using a field emission scanning electron microscope (FE-SEM, MIRA3 TESCAN) and a transmission electron microscope (TEM, Ziess EM900). With the use of X-ray energy diffraction spectrometry (EDS, MIRA3 TESCAN), elemental chemical characteristics were evaluated. The surface area was measured using the Brunauer–Emmett–Teller (BET, Belsorp mini II) technique, which included nitrogen adsorption–desorption at a temperature of 77 K. Meanwhile, the size of the pores and distribution of volume were examined using the Barrett–Joyner–Halenda (BJH) method. The electron–hole pair recombination was studied using the photoluminescence spectrum technique (PL, Varian Cary Eclipse). The transient photocurrent of the synthetic materials was determined using the Metrohm DropSens, μStat-i 400s. The absorption of 4-NP was measured using ultraviolet–visible spectroscopy (UV–vis) and the band gap energy was calculated using UV–vis diffuse reflectance spectroscopy (UV-DRS, Avantes-Avaspec-2048) in the range of 200–800 nm.

### 2.3 Synthesis of Polyaniline-*co*-polyindole (*x*% PANI–PIN)

**Figure 1:**
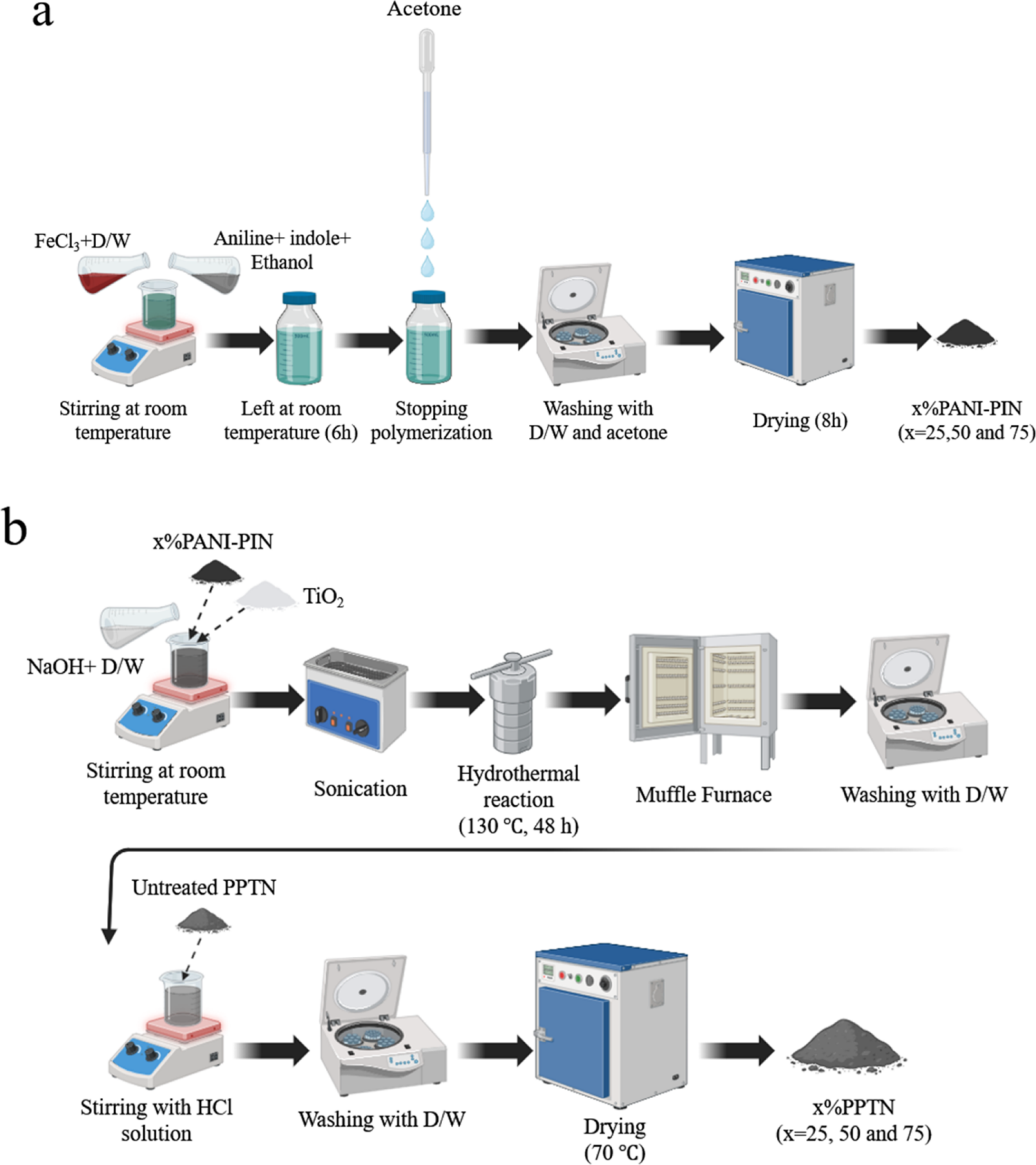
Schematic representation of (a) polymerization of *x*%PANI–PIN, (b) *x*%PPTN synthesis.

The production of this copolymer involved employing an approach rooted in existing literature, albeit with some adjustments.^30^ As shown in Figure 1a. Initially, 10 g of FeCl_3_ was dissolved in 100 mL of distilled water (D/W) designated as solution A. Subsequently, 1.3 mL of aniline and 0.58 g of indole were combined in 10 mL of ethanol (referred to as solution B). After achieving homogeneity through stirring, the two solutions were combined, stirred, and subjected to sonication for 1 h. The resultant mixture was maintained at room temperature for 6 h, following which 10 mL of acetone was introduced to halt the polymerization process. The product was then washed with distilled water and acetone and dried in an oven at 80 °C and 8 h. Similarly, pristine PANI and PIN were synthesized using the exact steps outlined above without the incorporation of the additional material. In this synthesis, “*x*” represents the proportion of PANI relative to PIN (*x* = 25, 50, or 75). For example, 75% PANI–PIN refers to a copolymer containing 75% PANI and 25% PIN.

### 2.4 Synthesis of PANI–PIN/TiO_2_ Nanotubes (*x*% PPTN)

To facilitate the creation of this composite, a straightforward hydrothermal technique has been employed. In Figure 1b, it is illustrated that, initially, 40 g of NaOH was dissolved in 100 mL distilled water, then 2.4 g of TiO_2_ powder was added to the solution. Afterward, 0.24 g of the as-prepared copolymer was added, stirred and sonicated for 1 h. This solution was transferred to a 120 mL Teflon-lined stainless-steel autoclave and heated at 130 °C for 48 h. Upon cooling of the autoclave, the formed PPTN was washed with distilled water several times. The resulting powder was then treated with a 0.1 M HCl solution under continuous stirring for 20 h. To reach a supernatant devoid of chloride ions, washing with distilled water was done and dried at 70 °C overnight.^35^ In this preparation, “*x*” in PPTN refers to the percentage of PANI in the PANI–PIN copolymer (e.g., 75%PPTN corresponds to the use of 75%PANI-25%PIN in the TNT).

### 2.5 Catalytic Activity Evaluation via Photocatalytic Degradation

**Figure 2:**
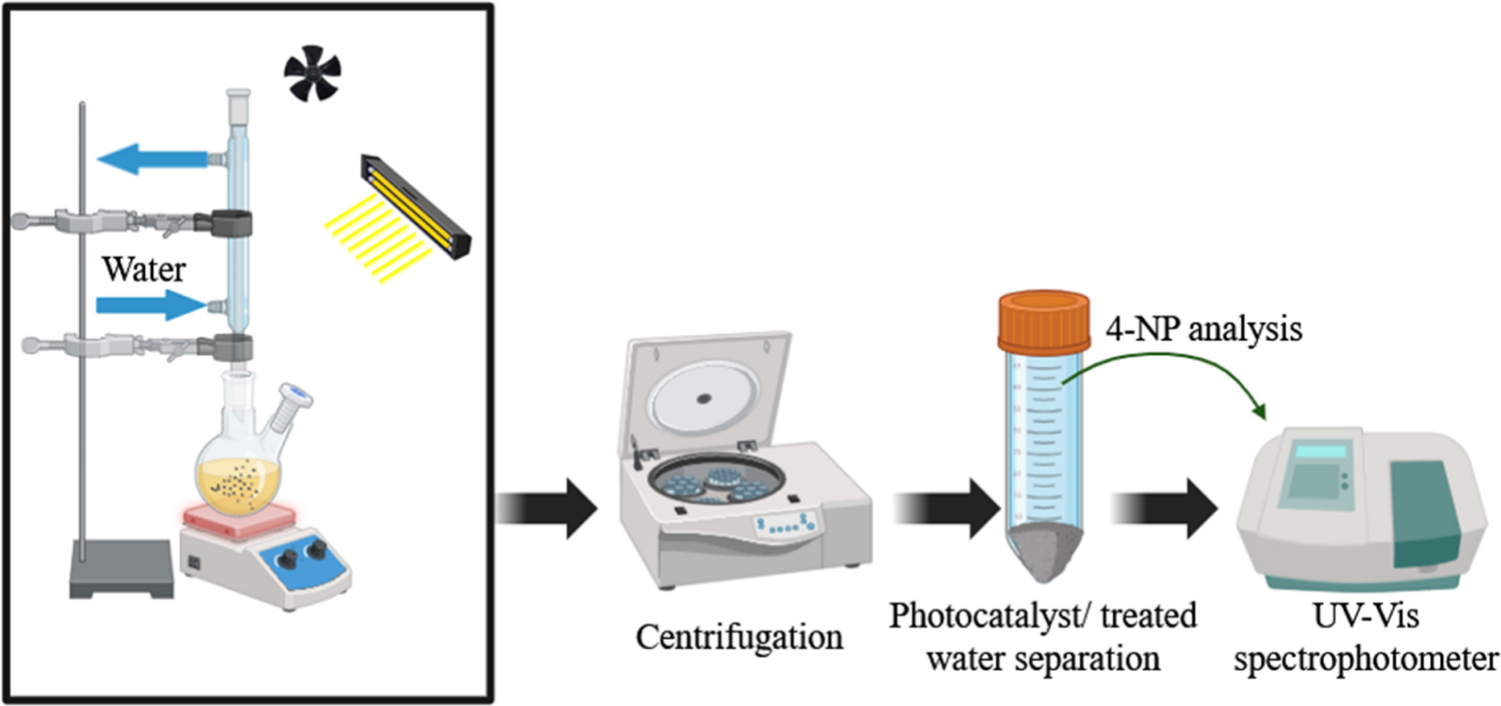
Scheme of the photocatalytic setup and photodegradation process.

To question the authenticity of the catalytic activity of the as-synthesized photocatalysts, 60 mg of the catalysts was added to 50 mL of 4-NP solution at a concentration of 10 mg/L. The initial solution pH was adjusted to 11 with 0.1 M NaOH solution. The photocatalytic tests were conducted in a reactor with a capacity of 250 mL. As exhibited in Figure 2, A condenser was positioned atop the reactor to condense any potential vapors by using water as a coolant. The reactor was fixed on a magnetic stirrer so that the yellow solution and the catalysts were kept under stirring. At first, this mixture was left in darkness for 30 min to reach adsorption–desorption equilibrium. The sampling was done to measure the amount of adsorption, after that the H_2_O_2_ was added and then visible light was applied and each 30 min the samples were taken. The solution’s yellowish color progressively turned colorless. To reckon the absorbance of taken samples, a UV–vis spectrophotometer was used, and the decline in the intensity of the absorption peak at 400 nm in 4-NP was monitored. *Y* = 0.1318*X* + 0.0321 is the resulted calibration line (*R*^2^ = 0.9995) through UV–vis device for 4-NP at a wavelength of 400 nm in which *Y* and *X* represent the absorbance of 4-NP and the concentration of 4-NP in the water in mg/L, respectively. Finally, at the end of the reaction, the catalyst and the solution were separated by employing centrifugation and the amount of 4-NP removal (NR) is determined via following equation
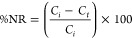
1where *C_i_* and *C_t_* are the concentration of 4-NP at the beginning and any specific time of the reaction in mg/L, respectively. The impact of influential parameters like time, pH, catalyst dosage, pollutant concentration, H_2_O_2_ and duration were investigated.

## 3 Results and Discussion

### 3.1 XRD Analysis

**Figure 3:**
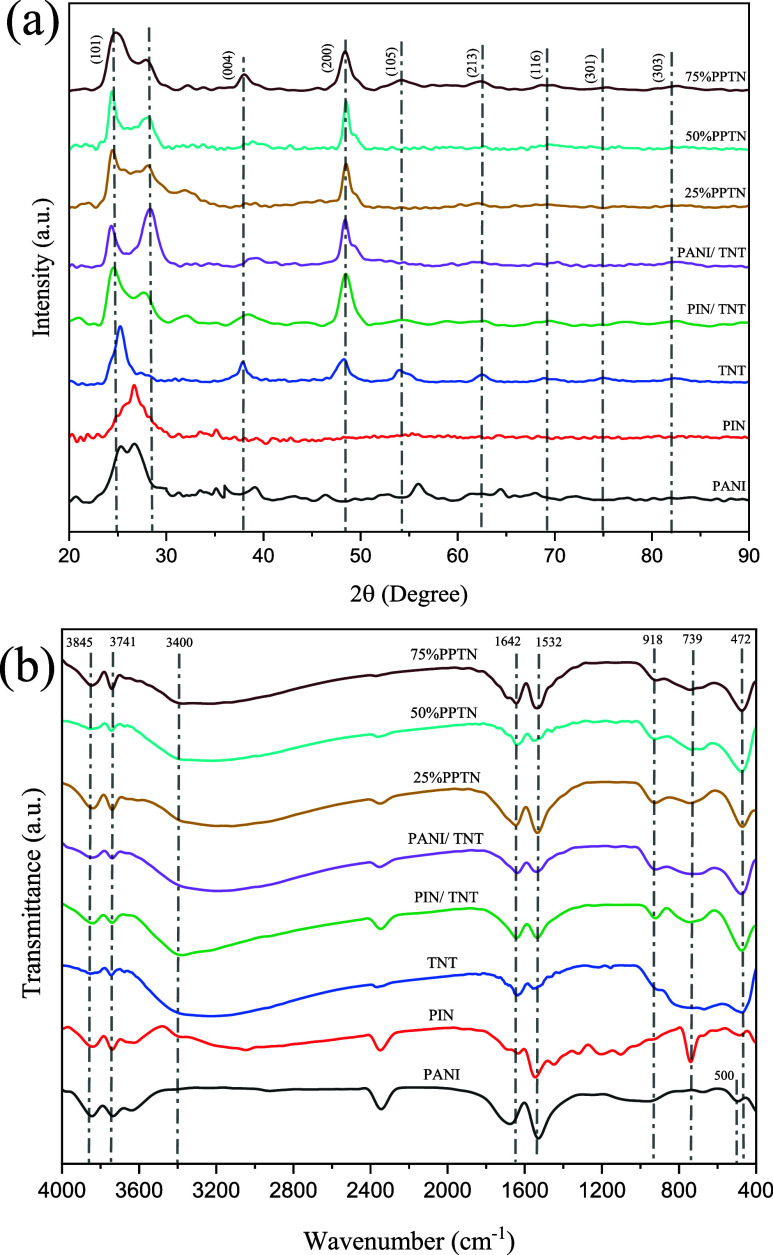
(a) XRD pattern and (b) FTIR spectra of PANI, PIN, TNT, PANI/TNT, PIN/TNT and *x*%PPTN samples.

The X-ray diffraction (XRD) pattern of PANI, PIN, TNT, PANI/TNT, PIN/TNT, 25%PPTN, 50%PPTN, and 75%PPTN samples was obtained in the range of 20 to 90°, as shown in Figure 3a. The presence of peaks in pure conductive polymers indicates the existence of their crystalline structure. The PANI sample had broad peaks at 25.3 and 26.7° which corresponds to its semicrystalline nature,^30,36,37^ whereas the PIN sample confirmed this with a broad peak at 26.6°.^38,39^ The peaks observed at angles of 25.3, 37.8, 48.3, 53.8, 62.47, 68.8, 74.9, and 82.48° in the TNT sample correspond to the crystallographic planes (101), (004), (200), (105), (213), (116), (301), and (303), respectively. These peaks reveal the presence of the anatase phase in the TNT sample, which is in agreement with previously published data in similar studies.^30,35^ A small peak at approximately 27.4° is observed, suggesting the existence of a rutile phase within the structure.^40^ Upon the addition of one or a combination of polymers, two distinct peaks are observed at angles of 25.3 and 48.3°. These peaks correspond to the (101) and (200) planes of the titanium dioxide anatase phase. The little reduction in peak intensity is ascribed to the incorporation of polymeric components, which affects the diffraction pattern owing to the diminished crystallinity of the composite and the encasement of TNT surfaces by polymers.^41^ The TiO_2_ phase has preserved its crystalline integrity, demonstrating the anatase phase despite the introduction of polymer.^15^ These findings point out that the addition of PANI, PIN, and their combination does not alter the crystalline structure of TNT, as seen by the FESEM and TEM micrographs provided below. In addition, although there is some overlap, the peaks of PANI and PIN can still be distinguished in the samples, confirming their synthesis and their placement in the composite. However, at an angle of 25.3°, there is overlap between the peaks of both PANI and TNT, the intensity of the peak indicates that the small polymer quantity may not be sufficient to fully mask or alter the anatase crystal structure.^35^ The size of the crystallite of a powdered material may be calculated through XRD analysis by applying the Scherrer equation to the XRD data.^42,43^ The efficacy of the photocatalyst gets affected by the particle size of the substance.^44^ It has been shown that smaller particle sizes result in a greater surface area to volume ratio, leading to enhanced photocatalytic functionality by facilitating better interaction between the photocatalysts and the pollutant.^30,44^ The Scherrer equation was used to calculate the average particle sizes in the present study.
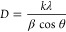
2

In this equation, *D* equals the average crystal size in nanometers, and *k* is the Scherrer constant, which here is 0.89. Additionally, λ represents the wavelength of the X-ray source, which in this case is 0.15406 nm (Cu Kα). β is the full width at half-maximum (fwhm) in radians, and θ denotes the peak location. Table 1 displays the average crystallite size and the percentage of crystallinity in the synthesized samples.

**Table 1:** Percentage of Crystallinity and the Average Crystal Size of Each Sample

samples	crystallinity (%)	average crystal size (nm)
TNT	31.1	5.74
PANI	40.1	18.28
PIN	10	2.19
PANI/TNT	25.2	8.17
PIN/TNT	30.3	8.39
25%PPTN	22.2	11.44
50%PPTN	18.6	11.05
75%PPTN	17.2	8.43

The noted reduction in crystallite size with increasing PANI content in PPTN samples can be explained by the function of PANI and PIN as capping agents, which restrict the growth of TiO_2_ crystals by wrapping and stabilizing the surface of the particles.^45^ Polymers such as PANI and PIN disrupt the processes of crystal nucleation and growth, leading to a decrease in particle size. The rise in polymer content enhances particle dispersion, effectively preventing agglomeration and further aiding in size reduction.^46,47^

### 3.2 FTIR Analysis

FTIR was used to analyze the functional groups contained in the synthesized samples. The findings are shown in Figure 3b. The peak detected at 472 cm^–1^ in the pure TNT sample corresponds to the bending vibrations of Ti–O–Ti. This peak is consistently observed in all samples synthesized with TNT, showing the existence of Ti–O–C bending vibrations. The existence of this bond in the TNT-synthesized composites is confirmed, resulting in enhanced light absorption of TNT in the visible region and subsequently minimizing its band gap. This finding is supported by data obtained from DRS.^4,35^ The intense vibrational peaks seen at around 3400 and 1642 cm^–1^ are caused by the stretching and bending vibrations of Ti–OH groups and adsorbed water molecules on the surface of the composite material.^35^ The peaks seen at about 500 and 739 cm^–1^ in the pure PANI and PIN samples may be attributed to the out-of-plane bending vibrations of C–H.^4,48^ Furthermore, in these two conductive polymers, a noticeable peak at about 1532 cm^–1^ is associated with the simultaneous stretching motion of carbon–nitrogen (C=N) quinoid. In addition, the comparatively low-intensity peak detected in the composite samples at around 918 cm^–1^ may be ascribed to the out-of-plane bending vibrations of C–H bonds.^30^ The peaks detected at roughly 1642 cm^–1^ in PANI and PIN samples may be attributable to the rearranged N–H bonds present in these samples. When combined with TNT, these bonds align at a similar wavenumber. Furthermore, the presence of N–H stretching bonds may be identified by evident peaks at 3400 cm^–1^ in all samples. The observed peaks corresponding to N–H bonds,^48^ Ti–OH bonds, and water molecules are all coincident at a single wavenumber, exhibiting overlap and differentiation among the samples. The peaks seen in the range of 3650–3850 cm^–1^ in pure PANI and PIN samples, as well as those including polymer and TNT, are ascribed to the presence of OH bonds that arise from the residual solvent.^49^

### 3.3 DRS Analysis

**Figure 4:**
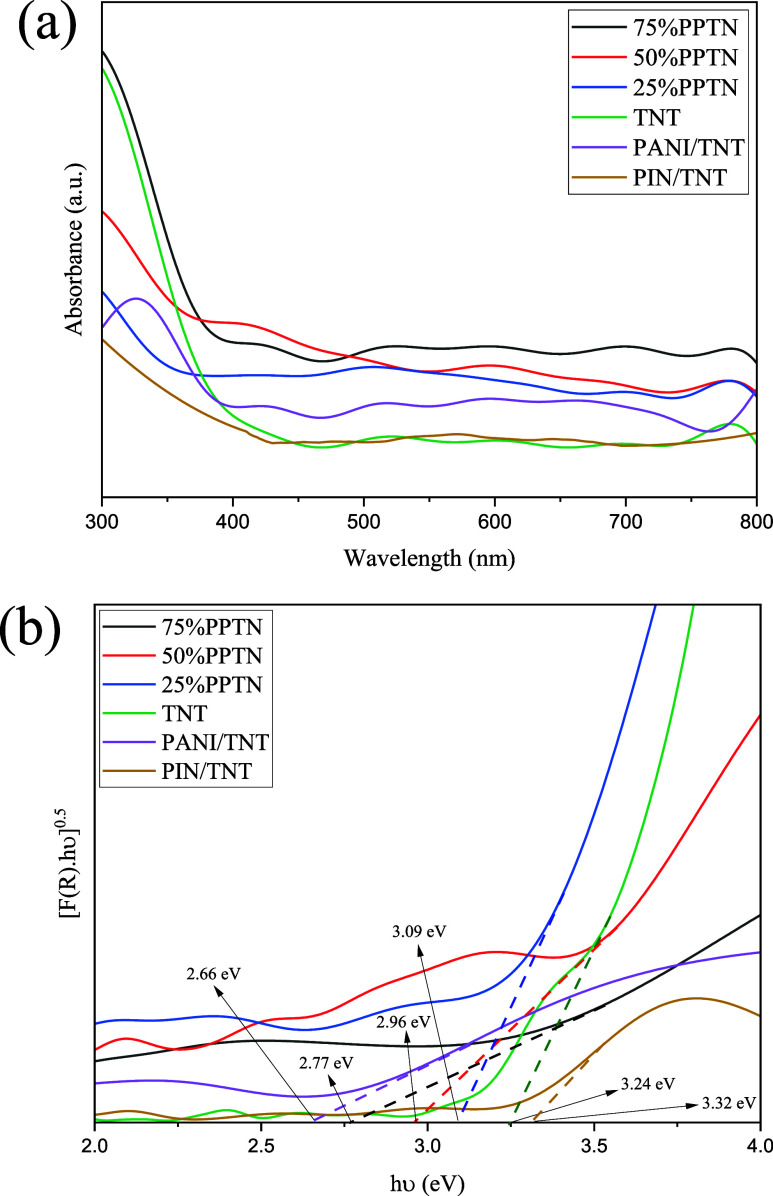
(a) UV–vis DRS spectra and (b) estimated band gap energies of TNT, PANI/TNT, PIN/TNT and *x*%PPTN samples.

The DRS technique was used to look into the optical characteristics of all samples. The DRS test findings have important implications in the early phases of photocatalytic processes, namely in measuring the photocatalyst’s ability to absorb photons. Put simply, the photocatalyst has to be able to absorb photons produced by the light source and then start the generation of free radicals, which are used to eliminate contaminants. Hence, it is essential to examine the optical characteristics of the produced photocatalysts. Figure 4a illustrates the light absorption capability of the synthesized photocatalysts within the wavelength range of 300 to 800 nm, serving the intended function. The graph clearly shows a drop in light absorption for the TNT sample at a wavelength of 400 nm, which is referred to as the absorption edge of this sample. Therefore, this sample lacks the ability to absorb light within the visible light spectrum and as a consequence, it will not be activated.^50^ This observation has been verified and validated by operational evaluation, the findings of which will be provided in later sections. Nevertheless, further samples demonstrate absorption in this particular location, suggesting the influence of doped conductive polymers in these composites. Put simply, the visible light range has been expanded for various composites in terms of light absorption. Alongside the findings discussed, PANI exhibits distinct benzoid (π–π*) and quinoid (*n*–π*) transitions, facilitating absorption in the visible spectrum, especially in the range of 400–600 nm. PIN demonstrates significant absorption of visible light attributed to π–π* transitions, thereby extending its photocatalytic capabilities. The electronic transitions in PANI and PIN enable a wider range of light absorption in comparison to TNT, which predominantly absorbs UV light because of its larger band gap (3.2 eV). This comparison highlights the combined effects of conductive polymers in broadening the absorption spectrum and improving photocatalytic efficiency.^51,52^ The sample with the maximum absorption intensity in the visible light region, known as the ideal sample with 75%PPTN, demonstrates superior effectiveness as a photocatalyst in performance testing, due in part to this factor together with other contributing variables.^39,53^ In contrast to other papers, the PANI/TNT sample produced in this study demonstrates absorption in the visible light range.^30^

Another key factor to take into photocatalysts is their band gap, which can be determined by analyzing the outcomes of the DRS test and using the Kubelka–Munk’s function
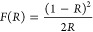
3
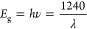
4where *R* is reflectance, *h* is Planck’s constant, ν is light frequency, *E*_g_ represents the band gap.^54^ The data shown in Figure 4b clearly demonstrates that 75%PPTN exhibits the lowest band gap (2.77 eV) in comparison to 25%PPTN and 50%PPTN. Based on the data from several sources, the findings of this study confirm that the band gap of TNT is 3.24 eV. Additionally, doping PANI has led to a considerable drop in the band gap, resulting in a lower energy need compared to comparable studies. The ideal sample exhibits a reduced band gap in comparison to the other two samples, suggesting an additional component that contributes to its superior performance in comparison to the others.^30,39^ The reduction in band gap observed with an increase in *x*%PPTN can be attributed to the improved conjugation and electron delocalization given by PANI. This enhancement promotes a more effective overlap of molecular orbitals, thereby facilitating charge transfer between PANI and TiO_2_. This results in a reduction of the band gap, since less energy is necessary for electronic transitions from the valence band to the conduction band. Moreover, the integration of PANI and PIN facilitates an enhancement in π-conjugation, thereby strengthening the mobility of charge carriers and optimizing the material’s light absorption within the visible spectrum.^55,56^ Additionally, the existence of Ti^3+^ may decrease the energy difference between the valence and conduction bands, so facilitating the separation of the photogenerated electron–holes.^57^

### 3.4 PL Analysis

**Figure 5:**
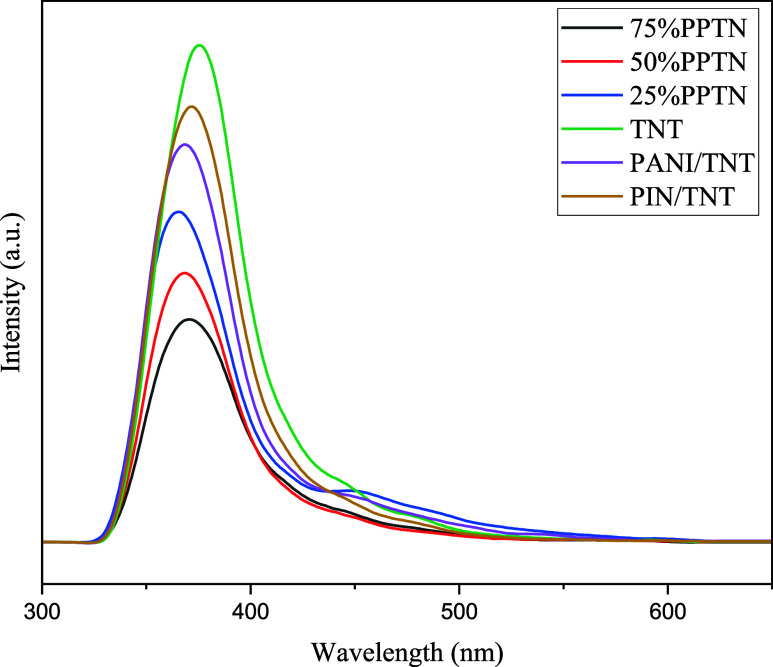
Photoluminescence spectra (PL) of TNT, PANI/TNT, PIN/TNT and *x*%PPTN samples.

A significant drawback of photocatalysts is the instability of photoexcited electrons transitioning from the valence band to the conduction band, resulting in a rapid rate of electron–hole recombination. Photoluminescence (PL) test findings are used to get data on the rate at which electrons and holes recombine.^30^ The intensity of the emission wavelength in a semiconductor sample is directly proportional to the rate at which electrons and holes recombine. In simpler terms, greater intensity corresponds to a higher recombination rate, which leads to worse photocatalytic efficiency.^58^Figure 5 depicts the photoluminescence spectra of the synthesized photocatalysts. All photocatalysts that have been synthesized have wide peaks within the wavelength region of 350 to 400 nm. As seen in the graph, the TNT sample exhibits a much larger peak intensity compared to the other samples. Moreover, the inclusion of PANI and PIN enhances the photocatalytic activity, resulting in a reduction in peak intensity. The 75%PPTN sample has the most minimal intensity peak. The phenomenon may be linked to the creation of a heterostructure, which effectively regulates the fast recombination of electrons and holes, facilitates the separation of charges, and improves the transportation of charge carriers. It is anticipated that 75%PPTN would perform better photocatalytically than other composites for the elimination of 4-NP because it achieves the lowest peak intensity in the PL spectrum. Heterogeneity enhances the process of charge separation, prevents recombination, enhances the movement of generated carriers, and enhances the photocatalytic activity.

### 3.5 Photocurrent Analysis

**Figure 6:**
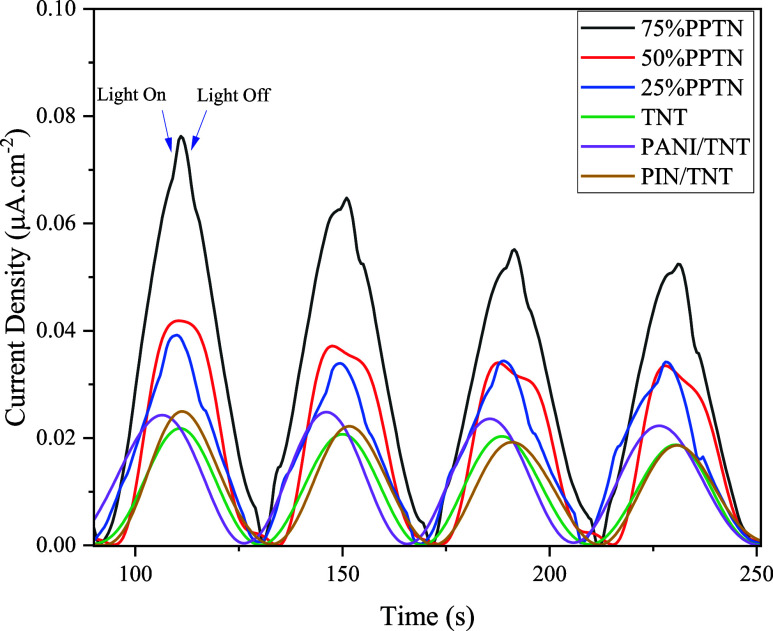
Transient photocurrent of TNT, PANI/TNT, PIN/TNT and *x*%PPTN samples.

Conducting photocurrent measurements is crucial for evaluating the photocatalytic characteristics of the synthesized composites. This experiment assesses the efficacy of electron–hole separation during light exposure, a vital component of photocatalytic performance.^59^ Efficient charge separation diminishes recombination and promotes the production of reactive oxygen species, essential for pollutant degradation. The photocurrent density measurements depicted in Figure 6 indicate a correlation with polymer content. The 75% PPTN composite has the strongest photocurrent response, reaching around 0.08 μA/cm^2^, while the bare TNT sample demonstrates the lowest response, hardly exceeding 0.02 μA/cm^2^. The rising photocurrent with elevated polymer content signifies that PANI and PIN facilitate charge separation and transfer, which are crucial for improved photocatalysis.^60^ Upon illumination, the photocurrent exhibits a rapid increase, succeeded by a sharp decrease upon cessation of light, signifying efficient creation and separation of electron–hole pairs. The significant reaction in the 75%PPTN composite underscores its enhanced charge transport characteristics, presumably attributable to a conductive polymer network. Augmented polymer content also amplifies visible light absorption, since PANI and PIN broaden the absorption spectrum, optimizing visible light use, which is essential for photocatalytic applications. The identified trend—maximum photocurrent at 75%PPTN, succeeded by 50% and 25%PPTN—substantiates the polymers’ contribution to enhancing photocatalytic efficiency.^61,62^ The findings demonstrate that the photocurrent density stays stable over time with minimal fluctuations in peak height across cycles, implying that the synthesized samples can consistently provide electrons and holes for the photocatalytic process.^59^ The diminished photocurrent responses from TNT, PANI/TNT, and PIN/TNT indicate that an inadequate mix of polymers results in insufficient charge separation and light absorption, highlighting the critical role of PANI and PIN in enhancing photocatalytic activity. In summary, the evidence indicates that raising PANI concentration in the polymer matrix results in enhanced photocurrent generation owing to superior light absorption and charge transport characteristics, hence improving the photocatalytic efficacy of the composite materials.^60,62^ This robustly substantiates the improved efficiency of the 75%PPTN sample in the photocatalytic degradation process.

### 3.6 BET Analysis

**Figure 7:**
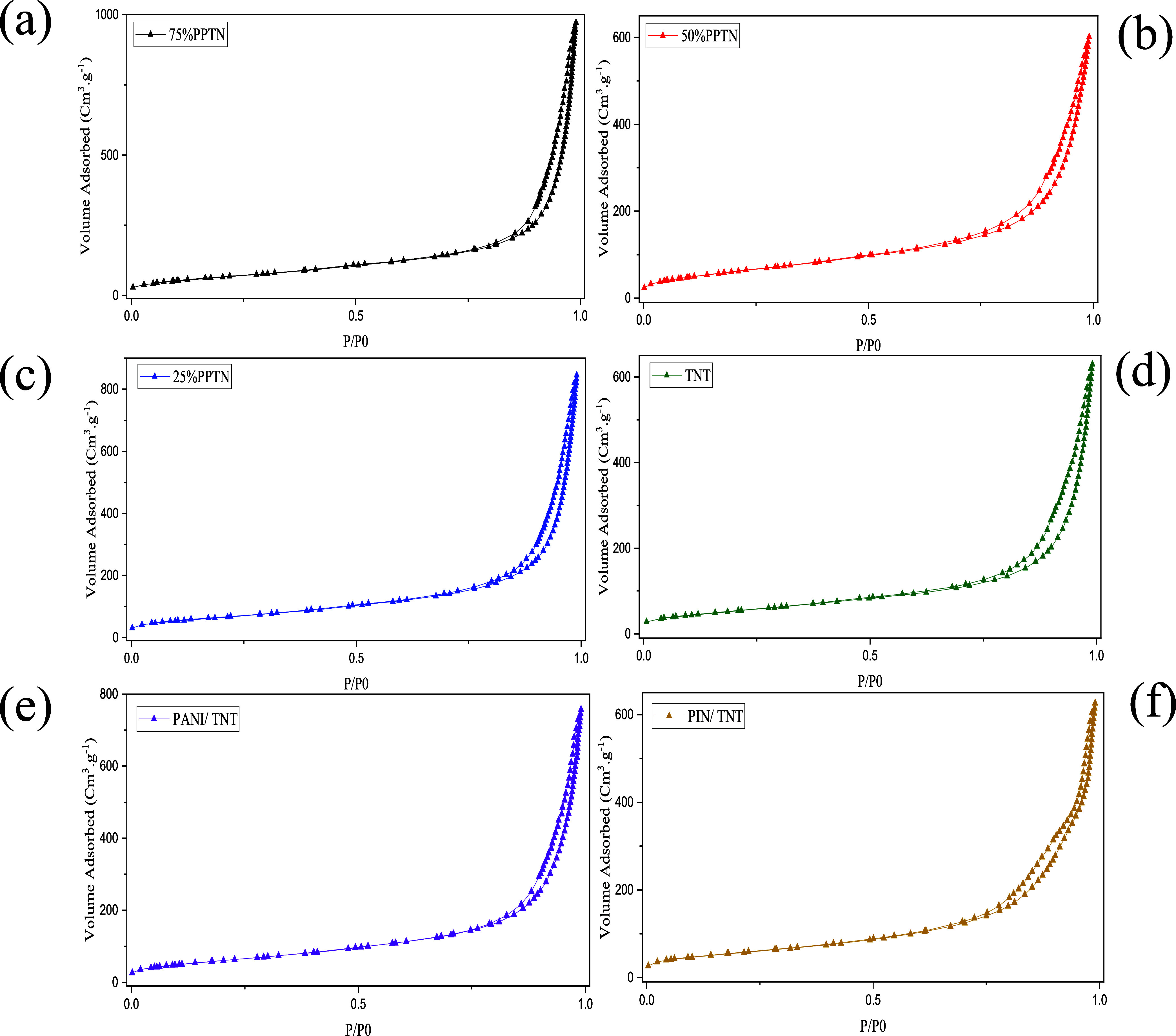
N_2_ Adsorption–desorption isotherms of (a) 75%PPTN, (b) 50%PPTN, (c) 25%PPTN, (d) TNT, (e) PANI/TNT, and (f) PIN/TNT.

**Figure 8:**
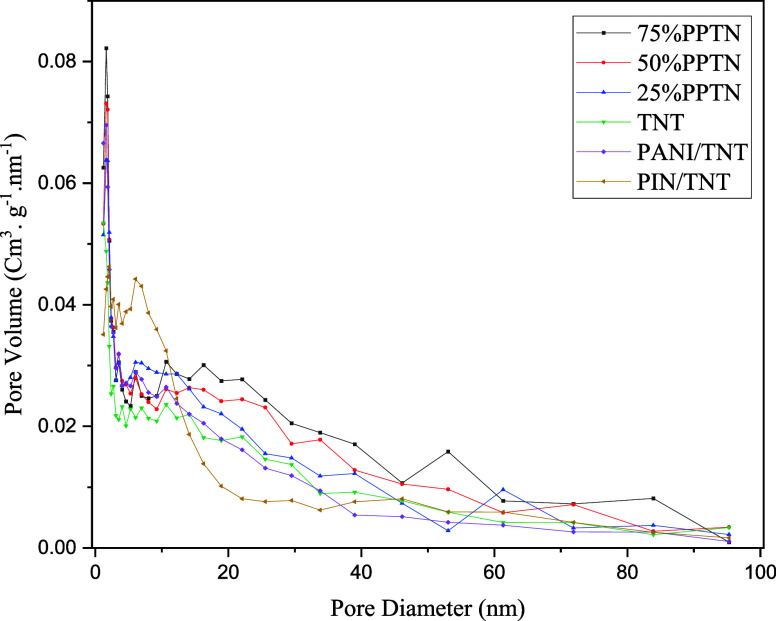
Pore size distribution (BJH) of TNT, PANI/TNT, PIN/TNT and *x*%PPTN samples.

Figure 7a–f displays the nitrogen adsorption–desorption isotherms, whereas Figure 8 illustrates the pore size distribution of the provided samples. Every as-synthesized photocatalyst falls under type IV of the IUPAC classification, which denotes a mesoporous structure. Furthermore, according to the categorization of hysteresis loops, they are classified as belonging to the H-3 category.^63,64^ The straightforward mechanism of mass transfer via mesoporous structures in photocatalysts has contributed to the widespread use of these structures.^30^ The accompanying chart clearly demonstrates that gas adsorption progressively rises from the most fundamental sample (TNT) to the optimal sample (75%PPTN). This factor results in an augmentation of the photocatalyst’s specific surface area, exposing a greater surface area to the pollutant 4-NP for the purpose of water purification. Table 2 presents three major output parameters obtained from the BET test: the specific surface area, the total pore volume, and the average pore size of each sample. These parameters align with the data obtained from the adsorption–desorption isotherm and performance tests. Based on the acquired data, TNT has the least specific surface area, measuring 199.1 m^2^/g. The introduction of polymers leads to an augmentation in the specific surface area, with measurements of 234.5, 237.5, and 255.3 m^2^/g for the three samples: 25%PPTN, 50%PPTN, and 75%PPTN, respectively. The sample with the greatest specific surface area has been designated as the ideal one. In addition, the total pore volume for all three samples exhibits an increase, with respective values of 1.16, 1.29, and 1.49 cm^3^/g. The 75%PPTN sample exhibits a substantial total pore volume and specific surface area, leading to considerably superior performance in comparison to the other samples. Put simply, the organic pollutant readily enters the mesopores of 75%PPTN and is adsorbed at its active sites.

**Table 2:** BET Specific Surface Area, Total Pore Volume, Average Pore Size and Optical Properties of TNT, PANI/TNT, PIN/TNT and *x*%-PPTN Samples

samples	*S*_BET_ (m^2^/g)	*V*_total_ (cm^3^/g)	*d* (nm)	*E*_g_ (eV)
TNT	199.1	0.9573	19.23	3.24
PANI/TNT	233.5	0.9246	15.57	2.66
PIN/TNT	202.4	0.9616	19.01	3.32
25%PPTN	234.5	1.1579	19.84	3.09
50%PPTN	237.5	1.2918	22.02	2.96
75%PPTN	255.3	1.4955	23.43	2.77

### 3.7 FESEM Analysis

**Figure 9:**
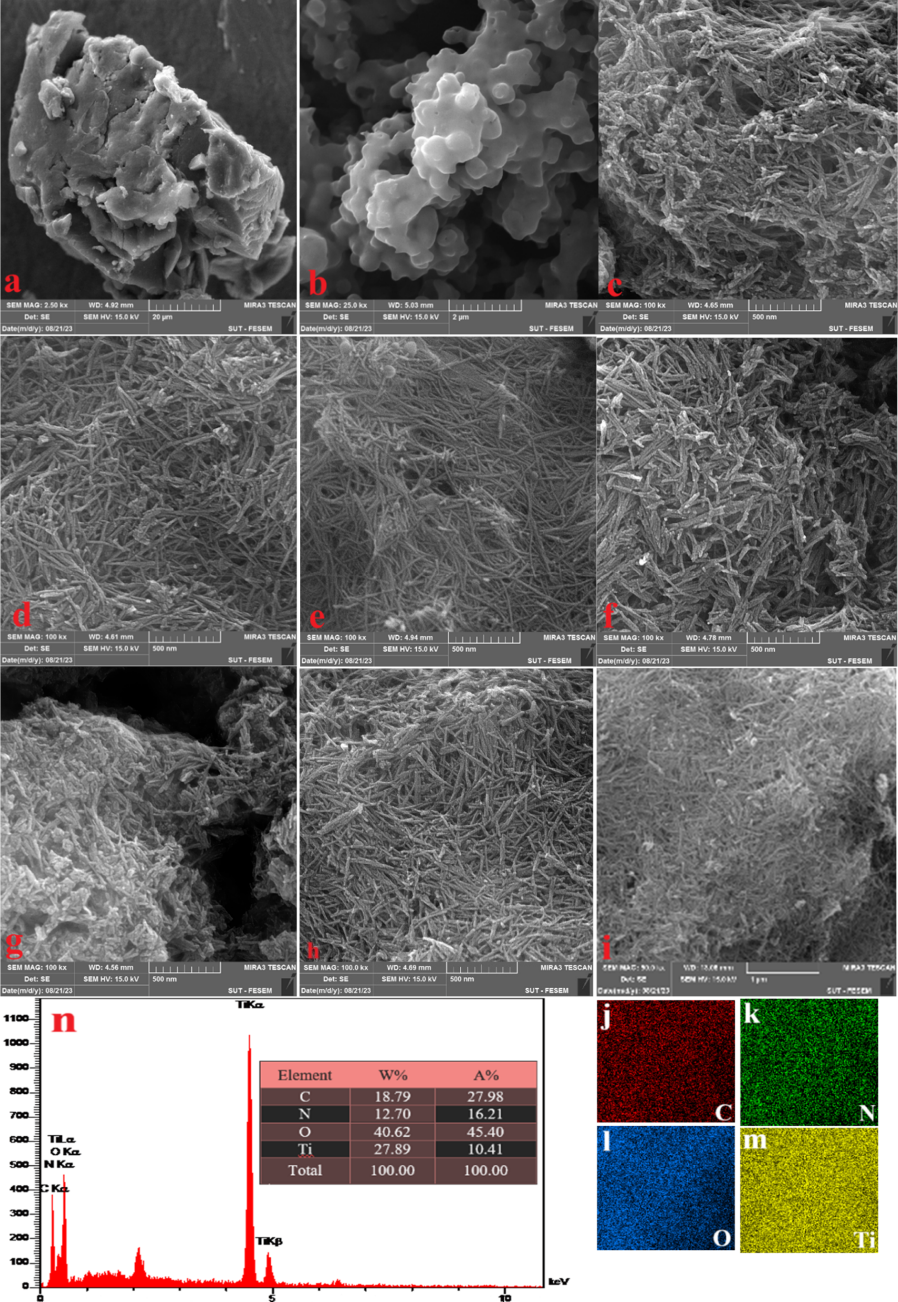
FESEM micrographs of (a) PANI, (b) PIN, (c) TNT, (d) PANI/TNT, (e) PIN/TNT, (f) 25%PPTN, (g) 50%PPTN, (h) 75%PPTN, (i–m) mapping of 75%PPTN samples and (n) EDX spectrum.

In order to get a more comprehensive assessment of the structure and form of the photocatalysts, we used FE-SEM analysis for all of the produced samples. Furthermore, Figure 9 also presents energy-dispersive X-ray spectroscopy (EDX) and mapping of the ideal sample, namely 75%PPTN. The first three pictures (Figure 9a–9c) depict the physical structure of the fundamental substances, which is PANI, PIN, and TNT. The polymeric structure of the polymers and the tubular shape of the produced TiO_2_ nanotubes, together with their porous nature, are easily discernible. The combination of one or both polymers with TNT results in the formation of porous nanotubes. These nanotubes have an average particle size ranging from 15 to 25 nm. The porosity of the 75%PPTN composite is larger compared to that of the 50%PPTN and 25%PPTN composites. This is attributed to the lower proportion of PIN employed in the 75%PPTN composite. The EDX and mapping analysis of the 75%PPTN sample revealed a uniform distribution of titanium, oxygen, nitrogen, and carbon elements. The weight percentages of these elements were found to be 27.89, 40.62, 12.7, and 18.79%, respectively. No impurities were detected in the sample.

### 3.8 TEM Analysis

**Figure 10:**
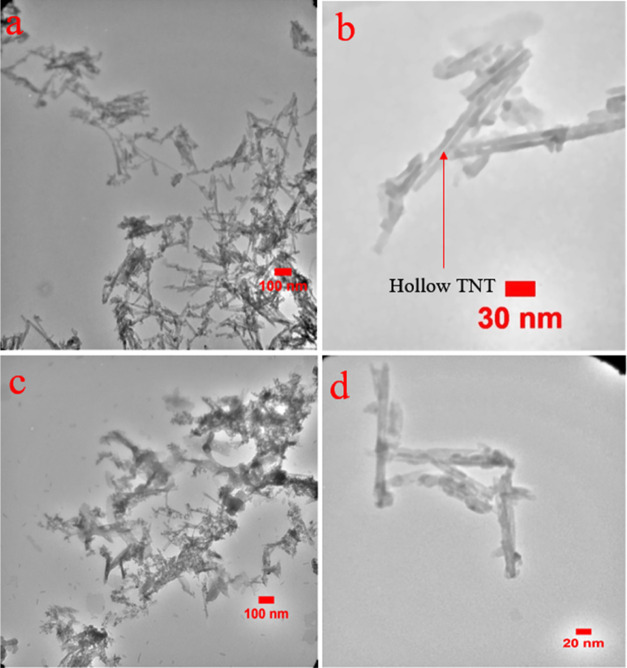
TEM micrographs of (a, b) TNT, (c, d) 75%PPTN samples.

To further analyze the morphology and structure of the synthesized nanophotocatalysts, transmission electron microscopy (TEM) images were obtained for both the TNT and 75% PPTN samples, as shown in Figure 10. The TEM images confirm the nanotubular structure observed in the FESEM images. For the pure TNT sample, a well-defined tubular structure with a hollow core is clearly visible, indicating empty space within the nanotubes. However, in the 75% PPTN sample, the presence of shadowed areas both within the nanotubes and along the outer tube walls suggests successful polymer coating. This indicates that the polymers have not only coated the exterior surface of the titanium dioxide nanotubes but have also penetrated the interior, filled the inner spaces and adhered to the internal walls.

### 3.9 Photocatalytic Experimental Tests

#### 3.9.1 Polymer Fraction Optimization on TNT

At first, to make sure of having photodegradation, some initial runs named as blank tests have been performed to analyze the key roles of the light source, photocatalyst itself, and oxidizer. The results are provided in the Supporting Information.

**Figure 11:**
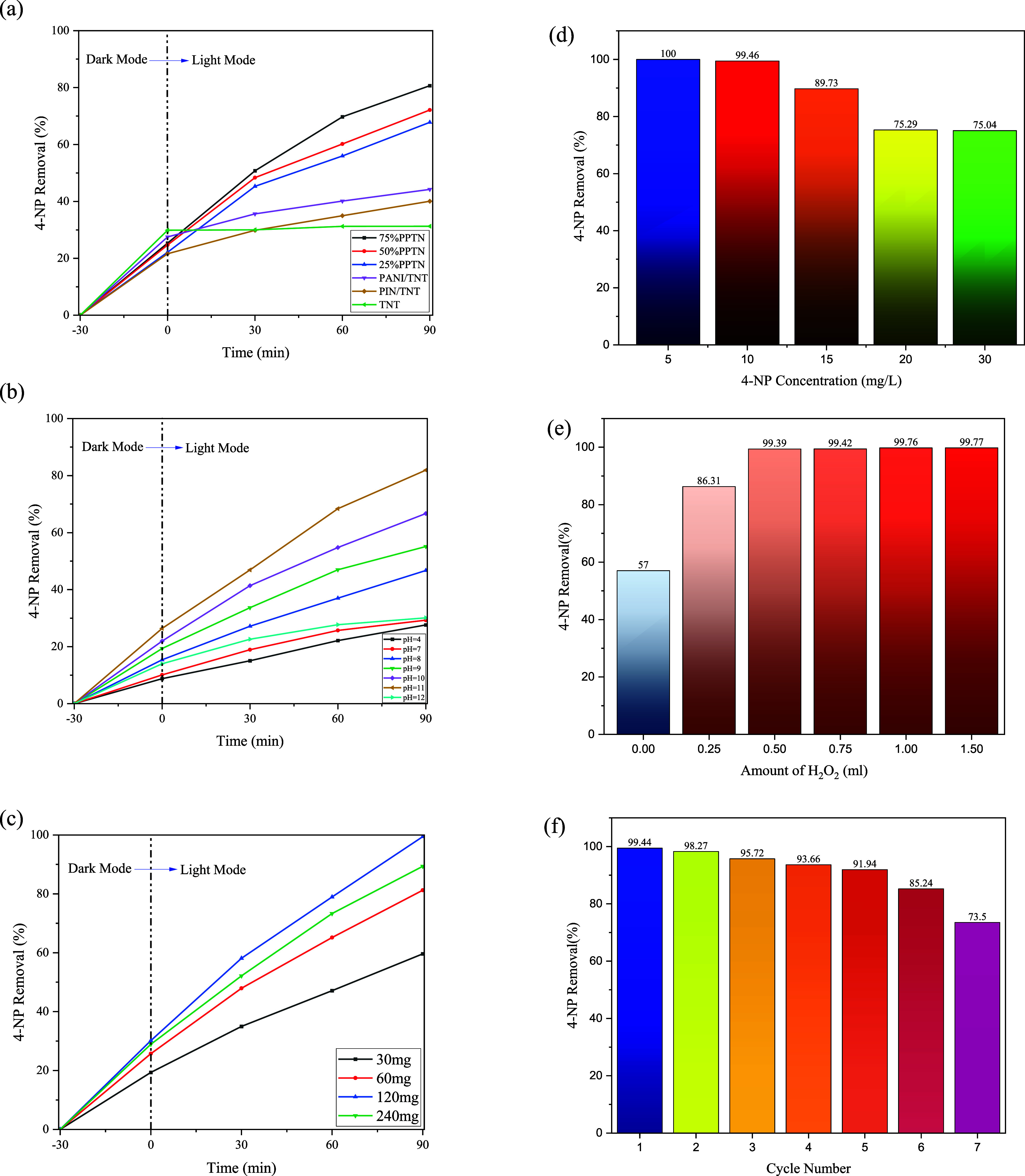
(a) 4-NP removal efficiencies of synthesized samples (*C*_4-NP_ = 10 mg/L, catalyst dosage = 60 mg, 0.5 mL H_2_O_2_ and pH = 11); monitoring the influence of (b) starting pH, (c) catalyst dosage, (d) 4-NP initial concentration, (e) oxidizer amount, and (f) successive reaction cycles on the catalytic performance of 75%PPTN (*C*_4-NP_ = 10 mg/L, catalyst dosage = 120 mg, 0.5 mL H_2_O_2_ and pH = 11 in every test, except mentioned).

To identify the most effective photocatalyst, we have synthesized six variant photocatalysts and compared them by applying them in experimental tests in similar conditions. Examining the results illustrated in the graph (Figure 11a), TNT demonstrated an overall 31.26% degradation of 4-NP, with 29.87% of this attributed to dark adsorption. This indicates that only 1.39% was due to photodegradation under visible light, aligning with the limitations imposed by TNT’s large band gap (3.2 eV), which requires UV light for efficient electron transfer. By the addition of PANI or PIN, we observed an increase in degradation and also the photocatalytic efficiency which means the facilitation of charge carrier transportation occurred due to these conductive polymers’ properties like their narrow band gap and high conductivity.^65,66^ Additionally, the TNT’s surface area is modified by treating with PANI, PIN and their combination, since it is in accordance with the results given by BET analysis.^30^ Alongside the mingle of PANI and PIN, a drastic soar in the removal was seen due to the synergic effects, the more PANI in the compound resulted in more efficient performance. With 80.68% removal, 75%PPTN illuminated the highest degradation compared to 25%PPTN and 50%PPTN, hence this compound was selected for the remaining experimental studies.

#### 3.9.2 Exploring pH Effect on 4-NP Photodegradation

Prior research has shown that the pH of the reaction solution is of paramount importance.^67^ This is due to the fact that it affects the adsorption–desorption processes of organic molecules on the surface of the photocatalyst.^30^ Not only that, but the formation of hydroxyl radicals besides the active species of oxygen at the photocatalyst’s surface is influenced by pH.^30,67^ Similar to previous sections, a 50 mL solution with a concentration of 10 mg/L was used at various pH levels. Additionally, 60 mg of the optimized catalyst and a light source were employed. Previous research indicated that the alkaline solutions were expected to have the highest performance, which is consistent with the findings shown in Figure 11b. Aside from conducting testing with alkaline solutions, other experiments were carried out using neutral and acidic solutions with pH levels of 4 and 7, respectively. As anticipated, these studies did not exhibit proper degradation. 4-NP has a negative charge in alkaline solutions, which is attributed to the presence of a phenolic group. On the other hand, the PANI and PIN surface possess a positive charge owing to their ion-exchange characteristics. Therefore, the active sites of the photocatalyst readily adsorbed 4-NP, contributing to a more advantageous occurrence of the photocatalytic activity in the alkaline solution.^68^ Consequently, the surface properties of the material resulted in the desired performance in basic solutions. The chart data clearly shows that as the pH increased toward neutrality, the degradation increased slightly from 27.63 to 29.3%. Despite this, starting from a pH of 8, the degradation progressively increased and reached 46.71%. The upward trajectory persisted until the pH reached 11, at which point it reached a maximum of 81.92%. The increase in pH triggered an increase in the removal of 4-NP. Nevertheless, once the pH reached 12, there was a decrease in the removal of 4-NP. This decrease was attributed to a highly negative photocatalyst surface, which hindered the production of hydroxyl and superoxide radicals. As a result, the light faced significant obstacles in reaching the surface of the photocatalyst. In other words, at a pH of 12, the concentration of hydroxide ions is significantly elevated, leading to a struggle between 4-NP and these ions for adsorption at the catalyst’s active sites. Consequently, a pH of 11 is selected as the optimum pH and used for further studies.

#### 3.9.3 Exploring Catalyst Dosage Effect on 4-NP Photodegradation

To assess the impact of catalyst dosage on the process, we conducted four experiments using different quantities of the optimized photocatalyst, namely 30, 60, 120, and 240 mg. The photocatalyst utilized in these tests was composed of 75% PPTN. All tests were carried out under identical circumstances. The topics pertaining to darkness, brightness, and the state of the light source being either on or off are entirely analogous to earlier examinations. Figure 11c demonstrates that when the catalyst quantity is increased from 30 to 120 mg, the removal efficiency of 4-nitrophenol rises from 59.61 to 98.12%. The boost in active sites has led to an increase in carrier separation, which is directly linked to the quantity of catalyst used.^69^ This is the primary explanation for the increase. When the parameter was increased to 240 mg, the removal effectiveness of the organic pollutant reduced from 99.43% (seen with 120 mg of catalyst) to 89.36%. An excessive rise in the quantity of catalyst brings about the clustering of catalyst particles and the creation of a catalytic aggregate, hence diminishing the surface area that is exposed to the pollutant (4-nitrophenol). Moreover, a higher concentration of catalyst hampers the transmission of light and diminishes the generation of carriers.^4,70^ Thus, based on the aforementioned considerations, a catalyst quantity of 120 mg has been selected.

#### 3.9.4 Exploring Initial Concentration Effect on 4-NP Photodegradation

To look into possible effects of the initial concentration of 4-NP on its photodegradation process, a concentration range of 5–30 mg/L was studied. In light of the preceding sections’ optimization, this investigation was carried out at a pH of 11 and with a catalyst dosage of 120 mg, using the optimized catalyst. The optimization results are shown in Figure 11d. Consistent with previous tests, the light source was turned off for the first 30 min, and the adsorption effect was observed. As anticipated, the initial concentration of 4-NP directly influenced the photodegradation process. Within 120 min, a solution with a concentration of 5 mg/L achieved complete removal (100%). Doubling the concentration to 10 mg/L did not lead to a substantial decrease, with a removal rate of 99.46% during the same time period. When the concentration was increased beyond 10 mg/L, there was a more noticeable declining trend. At 15 mg/L, the elimination of 4-NP decreased by around 10%, leaving 89.73% of the compound intact. At higher concentrations of 20 and 30 mg/L, the decrease persisted, and the percentage of removal remained almost constant. This phenomenon occurs as a result of the higher concentration of 4-NP molecules in the solution. These molecules are adsorbed onto the surface of the photocatalyst, causing interference with the path of photons that are supposed to reach the photocatalyst’s surface.^30^ Consequently, fewer hydroxyl radicals are generated, leading to a reduction in the efficiency of photocatalytic degradation.^71^ Based on the removal percentages, it can be concluded that the synthesized photocatalyst exhibits excellent performance even at high concentrations. In comparison to similar photocatalysts, it significantly outperforms them. For instance, at a concentration of 30 mg/L, the similar PANI/TiO_2_ catalyst was only able to remove approximately 45% of the pollutant.^30^ The acceptable performance may be ascribed to the existence of the secondary polymer PIN, which has enhanced performance as a result of its conductivity and optical characteristics.

#### 3.9.5 Exploring the Oxidant Amount Effect on 4-NP Photodegradation

The recombination of electron–hole pairs is a critical step that triggers energy loss and diminishes the effectiveness of photocatalytic degradation. This section examines the impact of one of the key factors on the performance of the photocatalyst by introducing varying quantities of H_2_O_2_. Throughout 6 test runs conducted under analogous circumstances, varying volumes of H_2_O_2_ (0, 0.25, 0.5, 0.75, 1, and 1.5 mL) were introduced to the reactor after 30 min of darkness, and at the moment the light turned on. The acquired findings are shown in Figure 11e. As demonstrated, the efficiency of the photocatalytic degradation reaction increases as the amount of H_2_O_2_ increases. However, it is important to note that beyond a certain threshold, the effect is minimized. This can be attributed to the saturation of active sites on the catalyst, which results in a lack of capacity for further absorption. Furthermore, an excessive quantity of H_2_O_2_ results in the poisoning of the catalyst surface. The elevated concentration of oxidizer inside the reactor leads to the consumption of hydroxyl radicals (^•^OH) by H_2_O_2_, resulting in a reduction in the overall concentration of radicals in the system.^71,72^ Based on the graph, the degradation rate is 57% when there is no oxidizer present. However, when 0.25 mL of oxidizer are added, the rate increases to 86.31%, suggesting the generation of optimal radicals. The upward tendency persists as the quantity of oxidant increases. However, in practical terms, the rate of elimination stays consistent after it surpasses 0.5 mL. Any amount over this threshold does not have an impact on the process of photocatalytic degradation. In order to avoid surface contamination and the depletion of radicals, it is recommended to use precisely 0.5 mL of H_2_O_2_.

#### 3.9.6 Exploring the Recyclability of Photocatalysts

An essential attribute of any photocatalyst, regardless of whether it is used in a laboratory or commercial configuration, is its exceptional stability for repeated reaction cycles. In order to achieve this objective, laboratory tests were carried out under ideal circumstances, which included using 50 mL of solution with a concentration of 10 mg/L, a pH level of 11, 120 mg of catalyst, and 0.5 mL of H_2_O_2_. After each cycle, the whole catalyst was isolated using a centrifuge, rinsed three times with ethanol, and then dehydrated at a temperature of 60 °C in order to ready it for the subsequent cycle. The findings of this inquiry are shown in Figure 11f, illustrating the outcomes achieved in seven successive cycles of 4-NP elimination. Clearly, over the first 5 cycles, the percentage of elimination was consistently above 90%. Even in the sixth cycle, it remains slightly below 90%, with a loss in efficiency of 14.2% compared to the first cycle. The seventh cycle exhibits a more pronounced deterioration in performance, with a decrease of 73.5%. Several factors contribute to the decline in efficiency, including catalyst degradation caused by reaction conditions and pollutants, as well as the adsorption of byproducts resulting from the decomposition of pollutants on the photocatalyst’s surface.^4,72,73^

The improved photocatalytic efficacy of the 75%PPTN nanocomposite is primarily due to its exceptional adsorption capability, significantly enabled by the selective interaction between the polymeric covering and the 4-NP contaminant. The conductive polymers PANI and PIN in the coating facilitate π–π stacking interactions and hydrogen bonding with aromatic molecules like 4-NP, hence enhancing adsorption efficiency substantially.^74^ Analogous to research on polymeric adsorbents such as poly(divinylbenzene-*co*-methyl acrylate), which exhibited improved adsorption of organic molecules via π–π interactions and selective dye adsorption based on molecular size exclusion,^74,75^ the customized architecture of the PANI–PIN-coated TNT facilitates effective adsorption of 4-NP onto the composite surface. This adsorption is essential since it maintains the pollutant molecules at the photocatalytic sites, guaranteeing their accessibility for subsequent breakdown under visible light irradiation.^74^ Moreover, the extensive surface area and consistent distribution of adsorption sites, similar to porphyrin-based porous organic polymers (Py-POP), boost the material’s capacity to absorb and decompose contaminants.^74^ The synergistic interaction between selective adsorption and photocatalysis facilitates the elevated degradation rates noted with the 75%PPTN composite, rendering it an exceptionally useful material for wastewater treatment applications.^74,75^

#### 3.9.7 Exploring the Reaction Kinetics

**Figure 12:**
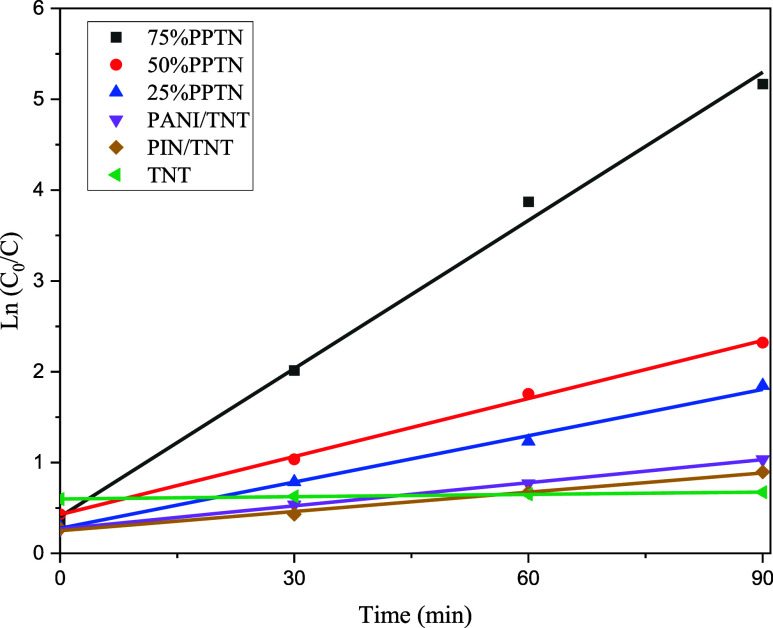
Obtaining the reaction rate constants by fitting the experimental data to a pseudo-first-rate model.

In this stage, we determined the rate constants for the synthesized photocatalysts using the experimental data collected under ideal circumstances. These conditions included a solution volume of 50 mL of solution with a concentration of 10 mg/L, a pH level of 11, 120 mg of catalyst, and 0.5 mL of H_2_O_2_. The collected data showed a strong correlation with a pseudo-first-order model. Thus, using eq 5, with *C*_0_ and *C* denoting the beginning and final concentrations respectively, *k* representing the rate constant of the reaction, and *t* indicating the time, we computed the reaction rate constants.^76,77^Figure 12 demonstrates that by graphing the curve versus time and then doing a linear regression on the data, the resulting slope corresponds to the response rate constant. The rate constants are shown in Table 3.
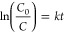
5

**Table 3:** Rate Constants of the Synthesized Photocatalysts

samples	*k* (min^–1^)	*R*^2^
TNT	0.0008	0.996
PANI/TNT	0.0085	0.9992
PIN/TNT	0.0071	0.9932
25%PPTN	0.017	0.9954
50%PPTN	0.0213	0.9981
75%PPTN	0.0544	0.9953

As indicated in the Table 3, the lowest rate constant belongs to TNT, followed by PIN/TNT. Then, with an increase in the amount of PANI, the reaction rate constant gradually increases, and for the optimized 75% PPTN sample, the highest rate constant value was 0.0544 min^–1^.

### 3.10 Proposed Mechanism for 4-NP Photodegradation

**Figure 13:**
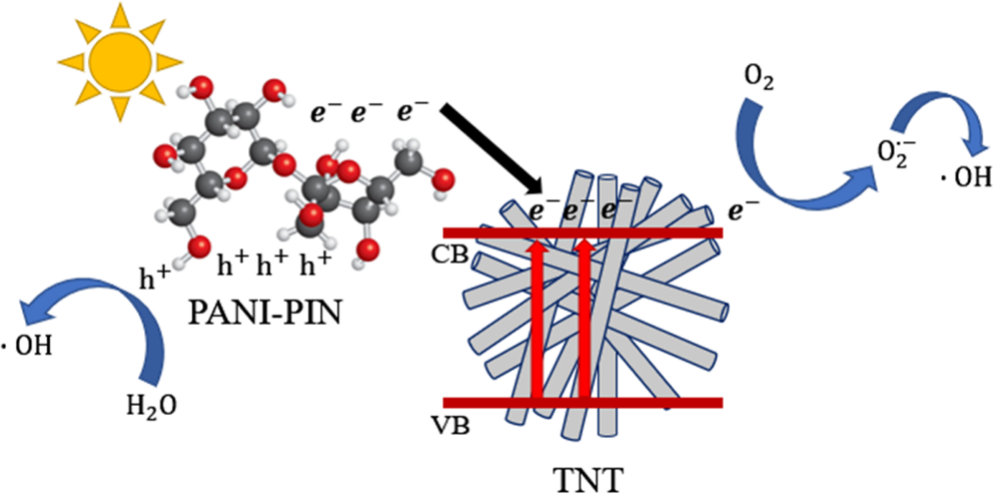
Proposed mechanism for 4-NP photodegradation process.

The proposed mechanism for the degradation of 4-NP is shown in Figure 13. The photocatalytic degradation of 4-NP by the 75%PPTN nanocomposite involves a series of reactions driven by the generation of photogenerated electron–hole pairs. Upon exposure to UV–visible light, the conductive polymer blend of PANI and PIN absorbs photons, resulting in the excitation of electrons from the valence band (VB) to the conduction band (CB). This photoexcitation creates electron–hole pairs, with holes (h^+^) in the VB and electrons (e^–^) in the CB of the PANI–PIN composite, as described in eq 6(73) The electrons in the CB of PANI–PIN are subsequently transferred to the CB of the TiO_2_ nanotubes (CB of TNT), which act as an efficient electron acceptor due to their lower CB energy level. This interfacial electron transfer is crucial for reducing electron–hole recombination rates, a common challenge in photocatalytic systems. By efficiently transferring the photoexcited electrons to the CB of TNT, the 75%PPTN nanocomposite maximizes the lifetime of the photogenerated carriers, thereby enhancing overall photocatalytic activity.^35^ Once in the CB of TNT, the electrons can participate in the reduction of molecular oxygen (O_2_) adsorbed on the composite surface, generating superoxide radicals (^•^O_2_^–^) through a one-electron reduction process (eq 8). These superoxide radicals are highly reactive oxygen species (ROS) and play a significant role in initiating the breakdown of organic pollutants like 4-NP.^67^ Simultaneously, the holes generated in the VB of PANI–PIN can oxidize water molecules, leading to the formation of hydroxyl radicals (^•^OH), as shown in eq 7. Hydroxyl radicals are known for their strong oxidative power, which is essential for attacking and degrading the 4-NP molecules. Moreover, H_2_O_2_, either introduced externally or formed in situ during the photocatalytic process, reacts with the superoxide radicals to produce additional hydroxyl radicals, as indicated in eq 9.^78^ This interplay between superoxide and hydroxyl radicals creates a synergistic effect, accelerating the degradation of 4-NP into intermediate products. These intermediates are subsequently further oxidized to form benign end products such as CO_2_ and H_2_O, as represented in eq 10.^79,80^ This stepwise mechanism is outlined as follows:

1. Photon absorption: 75%PPTN absorbs UV–visible light, exciting electrons (e^–^) and creating holes (h^+^)
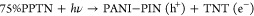
6
2. Oxidation of water molecules: The holes in the VB of PANI–PIN oxidize water, forming hydroxyl radicals (^•^OH)
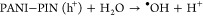
7
3. Reduction of oxygen: The electrons transferred to the CB of TNT reduce O_2_, forming superoxide radicals (^•^O_2_^–^)
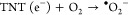
8
4. H_2_O_2_ and Reactive Oxygen Species (ROS) interaction: H_2_O_2_ reacts with superoxide radicals, forming additional ^•^OH radicals
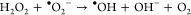
9
5. 4-NP degradation: Hydroxyl radicals attack 4-NP, leading to its breakdown into nontoxic byproducts

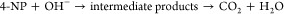
10

Furthermore, to enhance understanding of the ongoing processes, supplementary mechanistic insights are provided below:

1- Enhanced Charge Separation: The polymer coating of PANI–PIN on TNT enhances the visible-light absorption range and promotes charge separation by transferring photoinduced electrons from the polymer to the TNT.^81^ The spatial segregation of electrons and holes reduces recombination losses, a prevalent issue in photocatalytic devices.^26^ This elucidates the enhanced photoresponse of 75% PPTN.
2- ROS Formation: The generation of ^•^O_2_^–^ and ^•^OH is a well-documented pathway in photocatalytic degradation. The role of ^•^OH is particularly important, as it is one of the most reactive species in the oxidative degradation of organic pollutants.^82^ The ability of the 75%PPTN nanocomposite to generate a higher density of ROS, due to its large surface area and enhanced light absorption, is central to its high degradation efficiency.^83^
3- Synergistic Effects of PANI and PIN: The combination of PANI and PIN in the nanocomposite allows for better charge carrier mobility and stability. PANI, being a conductive polymer, provides a pathway for electron transport, while PIN enhances the photostability and chemical robustness of the composite.^66,83^ This dual functionality ensures that the photogenerated species remain active for a longer duration, contributing to sustained catalytic activity over multiple cycles.^35^

### 3.11 Comparison Study

Concentration, BET surface area, band gap, light source, and reaction time are some of the significant variables shown in Table 4 for eliminating 4-nitrophenol from wastewater. With its primary photocatalytic activity under visible light irradiation, 75% PPTN photocatalyst eliminates almost all 4-NP in 120 min. As a result, the majority of the photocatalysts described in the literature are inferior to the 75%PPTN photocatalyst.

**Table 4:** Evaluation of 75%PPTN’s Photocatalytic Capabilities in Relation to Those of Other Photocatalysts Described in Published Works

photocatalyst	4-NP concentration (mg/L)	*S*_BET_ (m^2^/g)	band gap (eV)	reaction time (min)	conversion (%)	light source	ref
Si-α-Fe_2_O_3_/CdS	20	56.78	1.73	120	66	incandescent lamp (160 W)	(84)
rGO/ZrO_2_/Ag_3_PO_4_			2.3	120	97	4 mercury lamps (6 W)	(85)
Au/ZnO	5		3.1	60	>95	xenon lamp (500 W)	(86)
GO-B-TiO_2_	25		2.9	180	100	tungsten lamp (100 W)	(6)
2D/3D Bi_2_MoO_6_/TiO_2_	50	54.29	2.82	180	95.3	xenon lamp (800 W)	(87)
Co-2B-TiO_2_	10	74.4	1.91	240	92	xenon lamp (150 W)	(88)
75%PPTN	10	255.3	2.77	120	99.44	sodium vapor lamp (250 W)	this study

## 4 Conclusions

This study presents the successful synthesis of titanium dioxide nanotubes (TNT) coated with conductive polymers, polyaniline (PANI) and polyindole (PIN), aimed at developing highly efficient photocatalysts for the degradation of 4-nitrophenol (4-NP) under visible light. The integration of these polymers into the TNT framework notably improved the photocatalytic performance, utilizing hydrogen peroxide (H_2_O_2_) as the oxidizing agent. The successful synthesis of the composite materials and the validation of their properties were confirmed through a series of advanced characterization techniques, including XRD, FTIR, UV–vis DRS, PL, transient photocurrent, BET, FESEM, EDX, and TEM. The composite comprising 75% PANI and 25% PIN (75%PPTN) exhibited outstanding photocatalytic performance among the materials evaluated. The distinct morphology of the nanotubes, combined with the conductive polymer blend, led to a decrease in electron–hole recombination, improved absorption of visible light, and a narrower band gap. The enhanced surface area of 75% PPTN significantly boosted its catalytic efficiency. Under optimal conditions—120 min of reaction time, pH 11, catalyst dosage of 120 mg, pollutant concentration of 10 mg/L, and 0.5 mL of oxidant—the 75%PPTN composite achieved an impressive 99.46% degradation of 4-NP. Furthermore, the 75%PPTN photocatalyst demonstrated exceptional stability and recyclability, sustaining over 90% degradation efficiency after five cycles of reuse. After six cycles, it maintained an impressive 85% degradation rate, highlighting its potential for sustained application. The results underscore the significant potential of 75%PPTN for effective wastewater treatment, showcasing its high efficiency, stability, and reusability for future large-scale or commercial applications.

## Supporting Information Available

Blank test procedures and analyses, including figures of the results (PDF)
