# Supplementary material for: Inside- and Outside-Coated PANI and/or PIN-TiO2 Nanotubes for Enhanced Photocatalytic Degradation of 4-Nitrophenol in Wastewater
Source: ACS Omega. 2024 Dec 16;9(52):51320–36. doi: 10.1021/acsomega.4c08137 (PMC11696435; doi:10.1021/acsomega.4c08137)
Supplement: Supplementary file 1 — ao4c08137_si_001.pdf [file ao4c08137_si_001.pdf]

Supporting Information

Inside- and outside-Coated PANI and/or PIN-TiO_2 Nanotubes for Enhanced Photocatalytic Degradation of 4-Nitrophenol in Wastewater

Seyed Mohammad Matin Ahmadi[a], Afsanehsadat Larimi[*,b], Ali Akbar Asgharinezhad[*,c], Farhad Khorasheh[a], Cyrus Ghotbi[a]

[a] Department of Chemical and Petroleum Engineering, Sharif University of Technology, Tehran, Iran
[b] School of Engineering and Applied Sciences, Department of Chemical Engineering, Swansea University, Wales, UK
[c] Chemistry and Process Research Department, Niroo Research Institute (NRI), Tehran, Iran

*Corresponding authors' Email: a.larimi@swansea.ac.uk (A. Larimi); aasgharinezhad@nri.ac.ir (A. A. Asgharinezhad)

Blank tests

The three components of light source, catalyst, and oxidant are significant and important in every process. Three separate experiments were conducted to evaluate the impact of the lack of each of these elements in this section. The first experiment investigated the impact of light and oxidant in the absence of the catalyst. The data shown in Figure. S1 clearly demonstrate that the absence of the catalyst leads to negligible changes and prevents the removal of pollutants. It is important to highlight that the test conducted without the catalyst does not include a 30-minute dark mode. In the second test, the reaction solution does not include any oxidant, and the catalyst facilitates the reaction in the presence of a light source. However, in this test, there is a 30-minute period without light, followed by the connection of the light source, and the reaction proceeds for a further 90 minutes. Clearly, when the oxidant is not present, the reaction efficiency is 57%. Out of this quantity, 29% is eliminated when exposed to light, while the remaining portion is taken in by the catalyst during the 30-minute period without light.

The previous experiment extensively examined the full absence of light from the source. It included two separate performance tests, one with the oxidant and one without. These experiments aimed to study the catalyst's absorption capacity and the impact of the oxidant on absorption. One crucial aspect of the photocatalytic process is that the catalyst becomes active when exposed to light and demonstrates suitable performance. To verify that the results obtained in the previous sections are not due to the catalyst's absorption and occur in the presence of light, we conducted two tests lasting 120 minutes: one without light and another with the presence of photocatalyst. Additionally, we performed a test with the presence of both photocatalyst and oxidant. Figure. S2 illustrates these tests. The efficiency of the first test, which measured the amount of absorption by the photocatalyst, was 25.5%. In the second test, where the photocatalyst was present alongside the oxidant, the efficiency reached 43.67%. The crucial observation is that absorption ceases after a consistent 30-minute period of darkness in both experiments. This cessation is due to the graph

reaching a stable state between 30 and 120 minutes, indicating no additional absorption taking place. As previously stated, the level of absorption has risen in the presence of the oxidant, suggesting a beneficial impact of this substance on absorption.

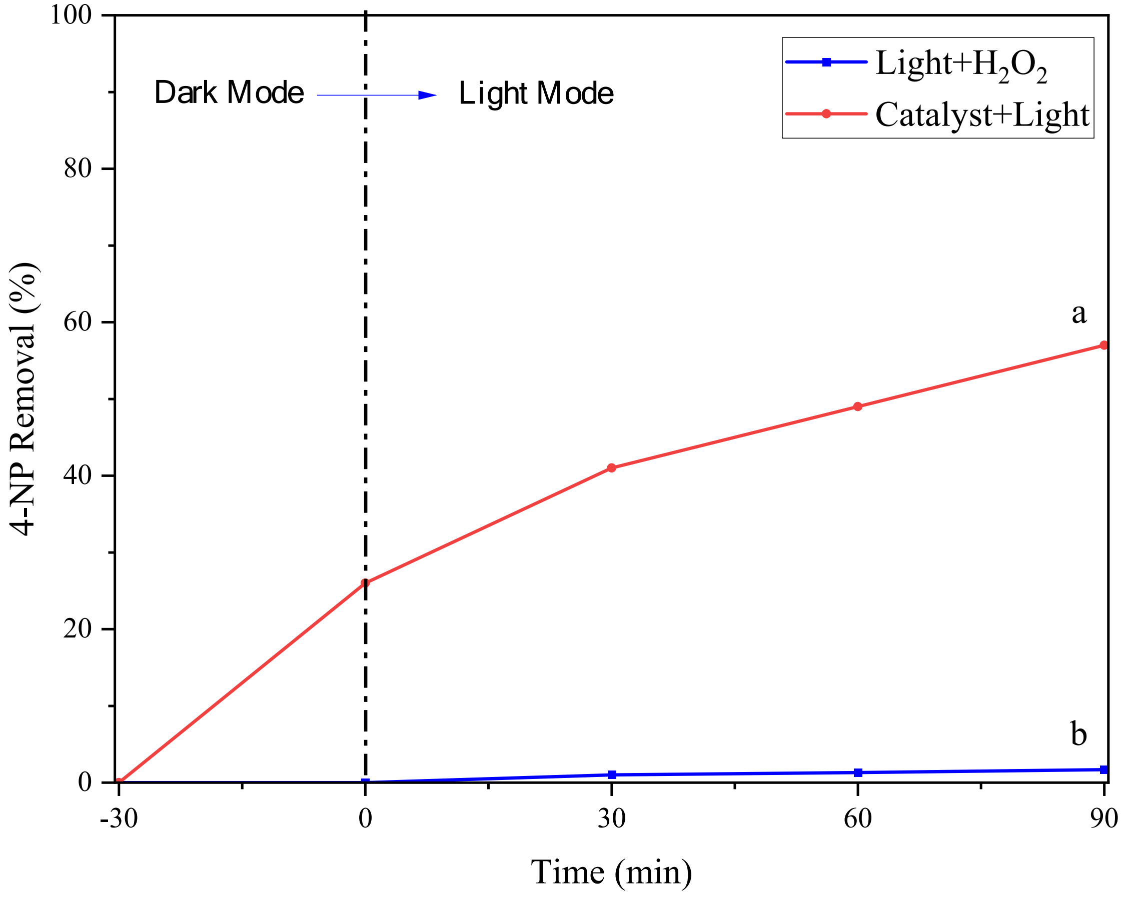

Figure. S1. Blank tests results (a) photocatalyst (75%PPTN) performance under visible light, (b) Oxidant efficiency in the absence of any photocatalyst

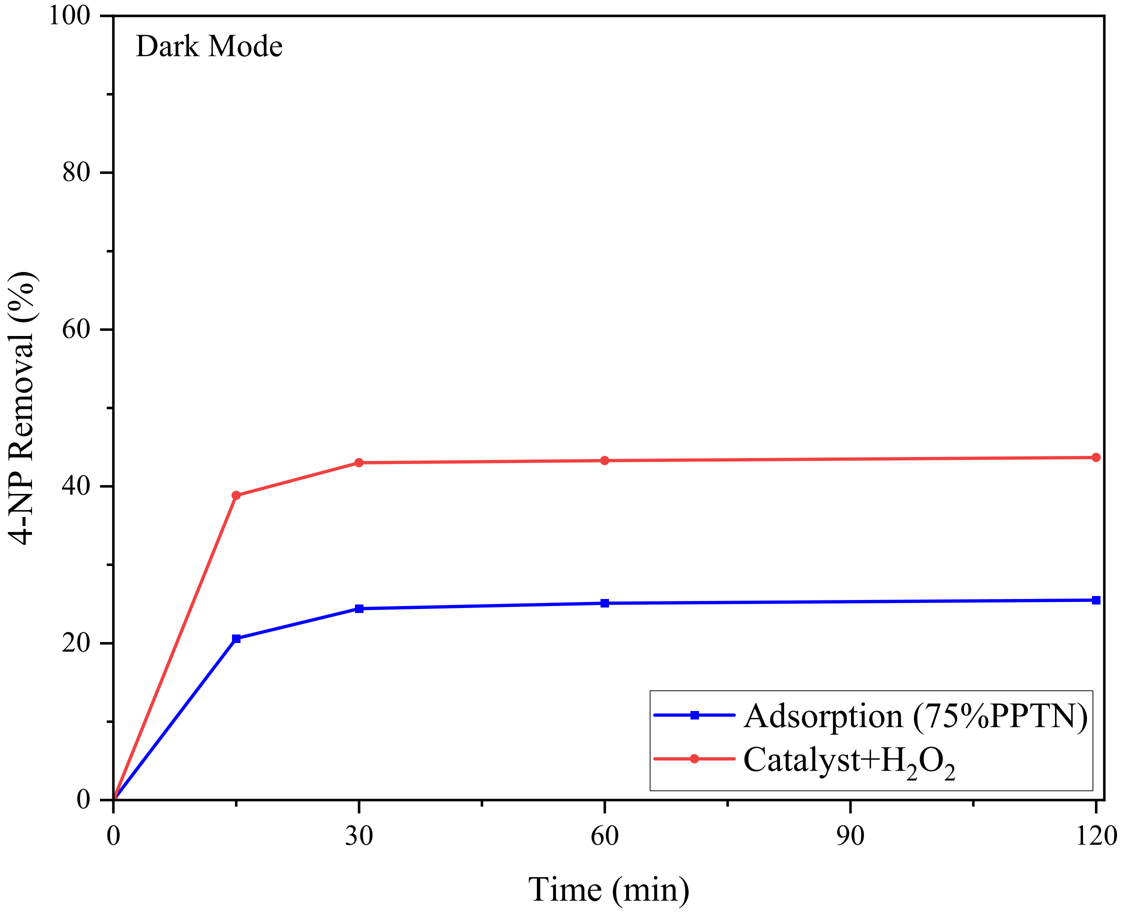

Figure. S2. Blank tests results of 75%PPTN adsorption with and without oxidant.
